# Identification of risk factors of acute coronary syndrome in young patients between 18-40 years of age at a teaching hospital

**DOI:** 10.12669/pjms.36.4.2302

**Published:** 2020

**Authors:** Faryal Murtaza Cheema, Hasan Mujtaba Cheema, Zubair Akram

**Affiliations:** 1 Faryal Murtaza Cheema. MRCP (UK)., Department of Medicine, Kishwar Fazal Teaching Hospital, Amna Inayat Medical College, Sheikhupura, Pakistan; 2 Hasan Mujtaba Cheema. MBBS., Department of Medicine, Kishwar Fazal Teaching Hospital, Amna Inayat Medical College, Sheikhupura, Pakistan; 3 Zubair Akram. FACA, FCPS (Cardiology).Professor and Head, Department of Cardiology, Jinnah Hospital, Allama Iqbal Medical College, Lahore, Pakistan

**Keywords:** Acute coronary syndrome, Cardiac enzymes, Risk factors, Young age

## Abstract

**Objectives:**

To identify the risk factors in acute coronary syndrome.

**Methods:**

It was a case series study, conducted in coronary care unit of Jinnah Hospital, Lahore from January to December 2018. Convenient sampling was used for patients’ selection. The serum cardiac enzymes level was measured, and serial ECG was done at admission and repeated if required. Blood samples were collected after an overnight fast of 14 hours and tests were done for total cholesterol and HDL cholesterol.

**Results:**

Out of 300 patients of acute coronary syndrome, 100 (33.33%) were female and 200 (66.67%) were males. Majority of patients 180 (60%) belonged to age group of 25-40 years. Out of 300 patients 94 (31.33%) had diabetes mellitus, while 139 (46.3%) were suffering from hypertension. Out of 290 patients 95 (32.7%) had family history of coronary artery disease. Out of 298 patients 125 (41.9%) were smokers.

**Conclusion:**

Acute coronary syndrome in age group of 18- 40 Years showed a male predominance with major modifiable risk factors; Hypertension followed by Diabetes mellitus, smoking and Dyslipidemia. Positive family history a non-modifiable risk factor in patients of ACS was also a common finding.

## INTRODUCTION

Acute coronary syndrome is a group of conditions caused by sudden blockage of the blood supply to the heart. It ranges from a potentially reversible phase of unstable angina to irreversible cell death due to a myocardial infarction. It constitutes an important problem because of the devastating effect of this disease on the more active lifestyle of young adults. It is now becoming a leading cause of death throughout the world.^1^ Acute coronary syndrome is one of the major causes of morbidity and mortality in Western European hospitals.^2^ It is also on rise in South Asian countries. It has been predicted that in future more than half of the worldwide cardiovascular disease burden will be borne by south Asian countries.^3^

There is limited data available in the form of formal studies from Pakistan. However small scaled studies conducted at different centers have looked into correlation of conventional risk factors with Coronary Artery Disease. A study conducted in Karachi, Pakistan showed that most commonly reported risk factor was dyslipidemia, followed by hypertension, diabetes, smoking and family history.^4^ Similar findings were reported from another study conducted at Pakistan Ordinance Hospital Wah, Pakistan.^5^ An increase in trend in the local population and involvement of younger ages has also been reported.^6^ Objective of current study was to identify risk factors in young patients with acute coronary syndrome admitted at a teaching hospital of Lahore.

## METHODS

This case series study included 300 patients (aged 18-40 years) who were diagnosed as case of Acute Coronary Syndrome (ECG and serum cardiac enzymes) and were admitted to coronary care unit Jinnah Hospital Lahore from January to December 2018. Detailed information and history of patients included in this study were recorded on separate Performa sheets. Informed consent was taken from every patient. The study was approved by the Ethical Review Board of Allama Iqbal Medical College Lahore Ref. No 52^nd^/ERB, dated 16 October 2019.

Patients diagnosed of ACS between age of 18-40 years with ST and Non ST elevated myocardial information and Unstable angina with electrocardiographic changes were included in the study. Patients of any other cardiac illness, pregnancy, stroke or cerebrovascular disease, stable angina, renal disease, liver disease and aged less than eighteen and more than forty were excluded.

ECG obtained and serum cardiac enzymes and other tests required were performed on admission and repeated whenever required. Instrument of data collection was developed which contained risk factors and variables of disease prognosis, socio-demographic risk, interventions and investigations. These data collection instruments were filled from the file of case history of the admitted patients. Data so obtained was analyzed by using SPSS version 21 to generate cross tabulation after frequency distribution. Results are presented in the form of percentages to meet standardization.

### Risk factors definitions

The study used following criteria to label the risk factors for the disease. History of sudden death or ACS before fifty-five years of age in father or male first degree relative for patients was considered to have a family history positive. Similar was the case when there was history of sudden death or ACS before sixty-five year of age in mother or female first degree relative. History of any tobacco smoking occasionally or daily and those who left this habit within a period of three months of diagnosis. Those on anti hypertensive medicines of any kind or with blood pressure more than systolic 140 mmHg and diastolic 90 mmHg at least on two occasions. Those on anti diabetic medicines of any kind or with blood sugar random more than 200 mg/dl or blood sugar fasting more than 126 mg/dl. History of high density lipoprotein less than 1.04 mmol/litre, low density lipoproteins equal or more than 3.37 mmol/litre and a total cholesterol more than 5.18 mmol/litre in patients either treated or diagnosed are labeled as patients of Dyslipidemia.^7^

**Table-I:** Information regarding risk factors for acute coronary syndrome.

S. No.	Risk Factors	No. of Patients	Percentage
1	Diabetes Mellitus	94	31.33%
2	Hypertension	139	46.3%
3	Dyslipidemia	97	20.6%
4	Family history of ACS	95	32.7%
5	Smoking	125	41.9%

## RESULTS

Out of 300 patients of acute coronary syndrome, 100 were females (33.33%) and 200 were males (66.67%). Most of the patients 60% belonged to the age group of the 25-40 years. The risk factors identified included dyslipidemia, diabetes mellitus, hypertension, family history of ACS and smoking. Ninety-four patients (31.33%) had diabetes mellitus and 139 (46.3%) were found to be hypertensive. A total of twenty out (20.6%) of ninety-seven tested for hyperlipidemia had high cholesterol and HDL levels. Family history of ACS retrieved from patients files, was positive in 95 (32.7%) and 125 (41.9%) had a history of smoking.

**Fig.1:**
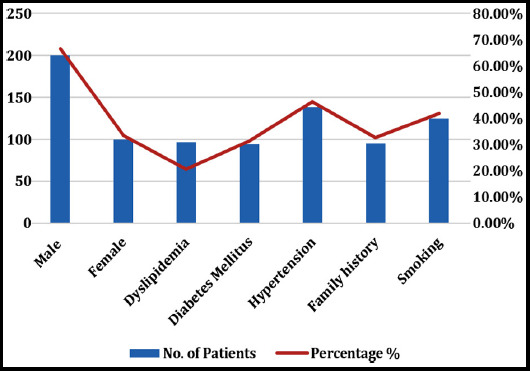
Sex distribution and the risk factors for acute coronary syndrome in 18-40 years of age.

## DISCUSSION

This study showed a male predominance (66%) with the involvement of modifiable risk factors; most common was hypertension (46%) followed by smoking (42%), family history (32%), diabetes mellitus (31%), and dyslipidemias (21%).

Results of similar studies from other centers of Pakistan showed high prevalence of modifiable risk factors as compared to this study. Major difference noted was dyslipidemia (91%) and (34%).^4^,^5^ The difference observed could be due to sample size as these studies had relatively small sample sizes compared to our study.

Risk factors frequencies disclosed by this study were similar to study by Tamrakar R et al.^7^ However the results of our study were not consistent with the findings of the study conducted by Akhtar et al. with respect to positive family history (57%) and dyslipidemia (63.2%).^8^ A study conducted by Balakrishnan et al. at tertiary care level in India showed high prevalence of all the risk factors; Male gender (83%) smoking (82%), hypertension (57%), diabetes (63%), dyslipidemia (66%), and family history (66%). Results of this study were not comparable with our study.^9^

In another study conducted by Jafary et al.^10^ the frequencies of risk factors revealed were male 68%, hyperlipidemia 18% and diabetes mellitus 37.2%, these findings were close to the findings of study under discussion. However, risk factors for ACS identified by our study were relatively different from another study conducted in Spain. That study showed that incidence of ACS among young patients was associated with diabetes and unhealthy lifestyle that included cocaine use, smoking, and obesity.^11^ Inconsistency observed in these studies might be due to difference in sample size, inclusion and exclusion criteria for the subjects of study.

Looking further into the details of individual risk factors unveiled by current study, it was observed that male predominance concurrence was found with almost all regional and international studies. Frequency of hypertension (46.3%) as risk factor of ACS was also in agreement with studies by Akhtar et al. (47.6%)^8^ and Gupta et al (33%).^12^ Hypertension in young adults with Acute Coronary Syndrome had also been implicated as independent risk factor for muti-vessel coronary artery disease.^13^ Patients of ACS also having chronic kidney disease exhibited very high prevalence of hypertension (81.3%) and diabetes mellitus (63.8%). It might be the consequence of chronic renal failure.^14^

Current study revealed that patients of ACS having smoking history were 41.9%. This finding was close to Gupta et al.^12^ (30%) but unlike to study by Singh et al (65%).^15^ Another study conducted in Pakistan by Muhammad F et al.^16^ showed frequency of smoking as risk factor for ACS as 46%, that was close to our finding of 41.9%.

A study conducted by Aram J et al.^17^ reported prevalence of family history and diabetes as risk factors of ACS, and were 24%, 20% respectively. Another study by Kumar V et al.^18^ showed prevalence of diabetes and family history as the risk factors of ACS/MI, 68.5%, 71.7% respectively. Findings of these studies and study under discussion were not consistent with each other’s. Reasons for these inconsistencies could be differences in sample size, inclusion and exclusion criteria for the studies.

Dyslipidemia is one important modifiable risk factor for acute coronary disease. Its prevalence in study under discussion was 20.6%. A study by Ricci B et al.^19^ about risk factors in ACS showed dyslipidemia 36.6%. Another study by Reda AA et al.^20^ about frequency of risk factors for ACS dyslipidemia was 38.5%. Possible explanation for these disparities’ other than reasons given above could be different lifestyles of the study groups.

ACS constitutes an important health problem because of the devastating effect of this disease on the more active lifestyle of young adults. As pointed earlier in the introduction that formal provincial/national data on the risk factors of disease is limited in Pakistan. Though this study was conducted in a resource-limited setting, however it is anticipated that it will add to existing quantum of knowledge regarding risk factors of the condition. Moreover, it will pave way for further multicenter studies and large level provincial or national survey to establish the baseline for modifiable risk factors.

### Limitations of the study

It was a single center case series study with small sample size and geographically limited. Identification of risk factor was based on the information available from the patients’ record. That might had been deficient or erroneous. This study analysed patients who reached the hospital, so it might not be a true representative of the population. Hence, the results cannot be generalized to the community.

## CONCLUSION

Hypertension, Diabetes Mellitus, Smoking, and hyperlipidemia were the major modifiable risk factors in our patients. However positive family history for ACS a non-modifiable risk factor studied was also a common finding. It is suggested that multicenter (hospital based) and provincial/national level community based studies employing standardized methodologies be conducted to establish the baseline for modifiable risk factors. This will enable in formulating policies for promoting healthy lifestyles, and age specific preventive strategies.

### Author’s Contributions

**FMC:** Conception and design. analysis and interpretation of data, Final approval of the version to be published She is also responsible and accountable for the accuracy or integrity of the work.

**HMC:** Data collection and drafting the article.

**ZA:** Reviewing the article critically for important intellectual content and general supervision of the research group.
